# Electrospinning of Fish Gelatin Solution Containing Citric Acid: An Environmentally Friendly Approach to Prepare Crosslinked Gelatin Fibers

**DOI:** 10.3390/ma12172808

**Published:** 2019-09-01

**Authors:** Anna Liguori, Jone Uranga, Silvia Panzavolta, Pedro Guerrero, Koro de la Caba, Maria Letizia Focarete

**Affiliations:** 1 Department of Chemistry “Giacomo Ciamician” and INSTM UdR of Bologna, University of Bologna, via Selmi 2, 40126 Bologna, Italy; 2 BIOMAT research group, University of the Basque Country (UPV/EHU), Engineering College of Gipuzkoa, Plaza de Europa 1, 20018 Donostia-San Sebastián, Spain

**Keywords:** fish gelatin, citric acid, electrospinning, pH, thermal treatment, gelatin structure, crosslinking degree

## Abstract

The majority of the crosslinking approaches employed to confer water resistance properties to electrospun gelatin mats are based on the use of potential cytotoxic agents, turning out to be not suitable for biomedical applications. Environmentally friendly chemical strategies based on the use of non-toxic agents are, therefore, strongly demanded. In the present work, the possibility to produce crosslinked electrospun fish gelatin mats by electrospinning an aqueous solution, containing citric acid as a crosslinking agent, is reported. The effect of pH on solution rheological properties, as well as on the electrospun mat morphology, chemistry, and crosslinking degree, is assessed. The increase of solution pH from 1.8 to 3.7 allows for obtaining fibers that maintain the fibrous morphology also in the mat. Subsequent thermal treatment of the electrospun mat (80 °C for 30 min) turns out to increase the crosslinking degree and morphological stability of the mat.

## 1. Introduction

Gelatin is a polymer obtained from the thermal denaturation or chemical degradation of collagen. These processes involve the loss of the collagen triple-helix structure and the formation of random coil structure typical of gelatin. Gelatin macromolecules can rearrange, under certain conditions, thus forming again sequences of the triple helix, even if the fibrillar collagen structure cannot be recovered and the material becomes highly soluble in an aqueous environment. Gelatin is employed in different fields, such as food industry, tissue engineering, medical applications [1,2,3,4], thanks to its biocompatibility, biodegradability, and low cost. Moreover, gelatin shows binding sites for cell adhesion, signaling, and differentiation, which make this polymer suitable in tissue engineering, wound dressing, and drug delivery [5,6,7,8,9]. In these sectors, electrospun nanofibrous mats are highly demanded, since they mimic the extracellular matrix and promote cell adhesion and proliferation due to their high porosity and surface area.

However, due to water solubility, gelatin nanofibrous mats do not maintain their morphology when they come in contact with water. In order to improve water resistance, physical [10] and chemical [11,12,13] crosslinking methods have been proposed. Focusing on chemical crosslinking approaches, agents such as genipin, diisocyanate, glutaraldehyde, and carbodiimide, have been successfully employed, being included in the gelatin solution prior to spinning [8,11,14,15,16]. Chemical methods also include post-treatment strategies based on the introduction of crosslinking agents after the spinning of the solution [13,16,17,18,19,20]. However, this approach demands a further step and, in some cases, has been reported to induce the flattening of the fibers, fusing them together [17,20]. Furthermore, some of the above-mentioned agents cause cytotoxicity [13,17] and are not suitable for applications in the medical field.

Citric acid is a natural acid and it has been already demonstrated to be suitable for the crosslinking of proteins [21,22,23,24]. The carboxylic groups of citric acid can undergo nucleophilic acyl substitution with the ε-amines of lysine, leading to the formation of stable amide bonds [21]. Saito et al. reported the use of a citric acid derivative, obtained through the modification of citric acid carboxyl groups with N-hydroxysuccinimide in presence of 1-ethyl-3-(3-dimethyl aminopropyl)carbodiimide hydrochloride, for the preparation of crosslinked type A porcine skin gelatin gels [25]. More recently, Shafagh et al. reported the use of citric acid to crosslink porcine skin gelatin in presence of Ag nanoparticles to produce, through a green approach in which water was used as a solvent, gelatin/Ag nanocomposite hydrogels with swelling and a drug release behavior both depending on pH [26]. Uranga et al. developed citric acid-crosslinked fish gelatin films, starting from an aqueous solution containing also glycerol and chitosan, with potentials for packaging applications since the presence of citric acid in the films did not produce any change of color, but improved both the light barrier and mechanical properties of the obtained films [27].

Among the different techniques for the production of polymeric substrates, electrospinning enables to develop micro- and nano-materials, and has been successfully used also to process fish-derived proteins [28], suitable for biomedical applications [29]. Citric acid has been used to crosslink collagen electrospun nanofibers. Cumming et al. successfully produced intra-fibrillar crosslinked marine collagen nanofibers by using the optimized citric acid: collagen molar ratio of 260:1 at pH 3.5 and performing a thermal treatment at 160 °C for 16 h after the mat fabrication [21]. Jiang et al. employed citric acid to produce crosslinked electrospun gelatin fibers using acetic acid as solvent and sodium hypophosphite as a catalyst of the citric acid, followed by a thermal treatment at 150 °C for 4 h to induce crosslinking [30].

To the best of our knowledge, no works have dealt so far with the production of crosslinked fish gelatin electrospun fibers by using an aqueous solution of citric acid, avoiding acetic acid or other chemicals. Indeed, only one previous work is present in the literature on the investigation of fish gelatin electrospinning in water/citric acid solution [31], but in this work the authors demonstrated that it was possible to obtain electrospun fibers only in the presence of additional acetic acid in the solution. Moreover, no studies or considerations are reported in this paper on the strategies to crosslink the as-produced mats.

**Scheme 1:**
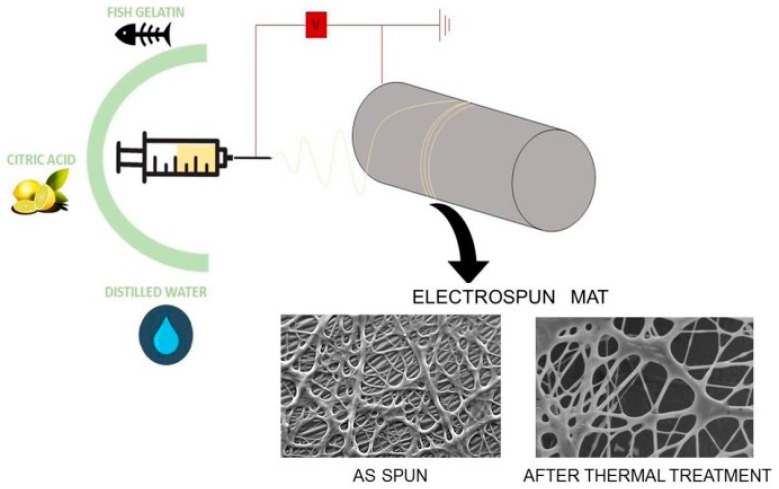
Scheme of the proposed approach to produce electrospun crosslinked gelatin fish fibrous mats.

The aim of the present work is to develop a protocol to make feasible, for the first time, the electrospinning process of fish gelatin by using only a natural and non-toxic crosslinker, such as citric acid, in aqueous solution to obtain crosslinked fibers, as illustrated in Scheme 1. The effect of the solution’s pH on electrospinnability and morphological and chemical properties of the resulting fibers was investigated. Furthermore, the effect of a subsequent thermal treatment of the mat on its morphological and chemical properties was also assessed.

## 2. Materials and Methods

### 2.1. Materials

240 bloom type A fish gelatin (FG), with an average molar mass of 125–250 kDa and a lysine content of 2.62%, was obtained from Healan Ingredients, North Newbald, UK, 280 Bloom type A gelatin from porcine skin (PG) was obtained from Italgelatine SpA, Via Statale, Italy. Anhydrous citric acid was provided by Panreac, Barcelona, Spain, sodium hydroxide, hydrochloric acid, 2,4,6-trinitrobenzenesulfonic acid (TNBS) and diethyl ether were purchased from Sigma-Aldrich, Milano, Italy. All chemicals were used as received without further purification.

### 2.2. Preparation of Solutions

FG solution was prepared by dissolving citric acid and gelatin in 3 mL of distilled water with citric acid/gelatin/water weight ratio of 0.9:1.0:3.0. The components were mixed together and kept at 50 °C under constant stirring (200 rpm) for 40 min. In order to modify the pH of FG solution, an FG+NaOH solution was obtained by dissolving citric acid and gelatin in 2.3 mL of distilled water with a citric acid/gelatin/water weight ratio of 0.9:1.0:2.3 and then adding 0.7 mL of NaOH 5 M. The compositions of FG solutions were optimized on the basis of rheological measurements, taking as a reference a PG solution. PG solution was prepared as previously described [8], in particular, PG was dissolved in a solution of acetic acid and distilled water (60/40 vol% acetic acid/distilled water) at a concentration of 30% (w/v). The obtained solution was stirred at 50 °C for 60 min. The pH of the solutions was measured by a calibrated pH meter (XS Instrument pH7, Carpi, Italy). In this work, the pH of the FG solution was 1.8, while the pH of FG+NaOH solution was 3.7.

### 2.3. Rheological Assessment of Solutions

The rheological measurements were performed using a rotational rheometer (Anton Paar MCR 102, Graz, Austria) operating in a plate-plate configuration. Experiments were performed keeping the temperature constant at 25 °C through the integrated Peltier system and a Julabo AWC100 cooling system, Seelbach, Germany. The sample was kept hydrated during the measurements through the use of a solvent trap (H-PTD200). Time-sweep oscillatory tests were carried out at a fixed strain amplitude of 0.3% and an angular frequency of 1 rad s^−1^

### 2.4. Electrospun Mat Fabrication

Mats were prepared by the electrospinning technique. The electrospinning apparatus consisted of a Spellman SL 50 P 10/CE/230 high-voltage power supply (Hauppauge, NY, USA), a KD Scientific 200 series syringe pump, and a glass syringe containing the polymer solution connected to a stainless-steel blunt-ended needle with an inner diameter of 0.51 mm through a PTFE tube. Electrospinning was performed at room temperature and relative humidity of 40–50%. FG solution was electrospun by using the following conditions: applied voltage = 20 kV, feed rate = 0.3 mL h^−1^, and needle-to-collector distance = 15 cm. The electrospinning of FG+NaOH solution was carried out using the same parameters employed for FG solution, except for the voltage and flow rate which were increased up to 23 kV and decreased up to 0.1 mL h^−1^, respectively, due to the higher solution viscosity conferred by NaOH. After fabrication, some mats were subjected to a thermal treatment carried out at 80 °C for 30 min under vacuum. The obtained mats were stored in a desiccator at 4 °C until further analysis.

### 2.5. Characterization Methods

A polarized optical microscope (OM) (Zeiss Axioscop, Zaventem, Belgium) was used to detect electrospun fibers directly collected on glass slides during electrospinning. Scanning electron microscope (SEM) observations were performed 24 h after mat fabrication at an acceleration voltage of 15 kV. Samples were mounted on a stub with double-side adhesive tape and sputter-coated with gold before observation. The distribution of fiber diameters (average and standard deviation) was measured on the SEM images of about 50 fibers by means of ImageJ software. Wide-angle X-ray diffraction (WAXD) analysis was carried out using a PANalytical powder diffractometer (Almelo, Netherlands) endowed with a fast X’Celerator detector. The radiation was generated from a CuKα (λ = 0.15418 nm) source (40 mA, 40 kV). WAXD data were obtained from 2θ values from 5° to 60°, where θ is the incidence angle of the X-ray beam on the sample. Fourier transforms infrared (FTIR) spectroscopy was carried out on a Nicolet 380 FTIR spectrometer (Thermo Scientific, Waltham, MA, USA) using ATR Golden Gate. 32 scans were performed with a resolution of 4 cm^−1^ in the range 4000–800 cm^−1^. Spectra were smoothed using the Savitzky–Golay function and second-derivative spectra of the amide I region were used at peak position guides for the curve fitting procedure, using OriginPro 9.1 software.

### 2.6. Crosslinking Extent

The crosslinking extent was measured according to the method of Panzavolta and coworkers [8]. Briefly, an UV assay of uncrosslinked ε-amino groups was performed on differently treated mats and on fish gelatin as reference. After the reaction with 0.5% TNBS, gelatin was hydrolyzed with 6 M HCl and extracted with diethyl ether. The solution’s absorbance was measured against a blank at 346 nm. The moles of free Ɛ-amino groups per gram of gelatin were calculated by the following Equation (1):

(1) $$\mathrm{Moles}\;\mathrm{of}\;\varepsilon –\mathrm{amino}\;\mathrm{groups}/g\;\mathrm{of}\;\mathrm{gelatin}\;=\;\frac{2\cdot A\cdot V}{\varepsilon \cdot b\cdot x}$$

where *A* is the sample absorbance, *B* is the final sample volume (L), *Ɛ* is the TNP-lys molar absorptivity (1.46 × 10^4^ L mol^−1^ cm^−1^), *b* is the cell path length (cm), *x* is the sample weight (g).

The cross-linking extent (CE) was determined from the ratio between the moles of crosslinked ε–amino groups of treated gelatin mats (obtained as a difference between uncrosslinked groups before and after crosslinking) with respect to *ε*–amino groups measured in fish gelatin.

## 3. Results and Discussion

### 3.1. Electrospinning of Fish Gelatin

It has been widely reported that the addition of an acid to water solutions, or the use of solvents such as 1,1,3,3,3-hexafluoro-2-propanol, is required for gelatin electrospinning to prevent gelation that hinders and even blocks the solution flow through the syringe needle and capillary during the spinning process [32,33]. Due to the possible toxicity of the most employed acids or organic solvents, efforts have been devoted to the fabrication of gelatin electrospun fibers using solutions of benign acids in water. Acetic acid, malic acid, and citric acid water solutions, as binary, ternary, and quaternary solvents, were recently used to manufacture FG based electrospun mats [31,32]. Although positive results on the feasibility of the process were achieved for water/acetic acid solvents, citric acid based binary solvent was reported to be non-suitable for developing fibers [31].

Considering both the absence of toxicity of citric acid and its potential crosslinking action, in this work efforts have been carried out to identify a procedure for the production of crosslinked FG electrospun mats using a spinning solution containing only citric acid in an aqueous solution. In particular, the effect of solution’s pH on the electrospinning process, mat properties, and crosslinking degree was investigated by adding NaOH to the FG citric acid/water solution in order to increase the pH of the FG solution from 1.8 to 3.7, on the basis of a previous study on collagen electrospun fibers crosslinked with citric acid [21]. In the mentioned paper the highest crosslinking extent was achieved from a solution containing collagen and citric acid with a pH 3.5, a result that was attributed by the authors to the more effective formation of citric anhydride at this pH [21,34].

**Figure 1:**
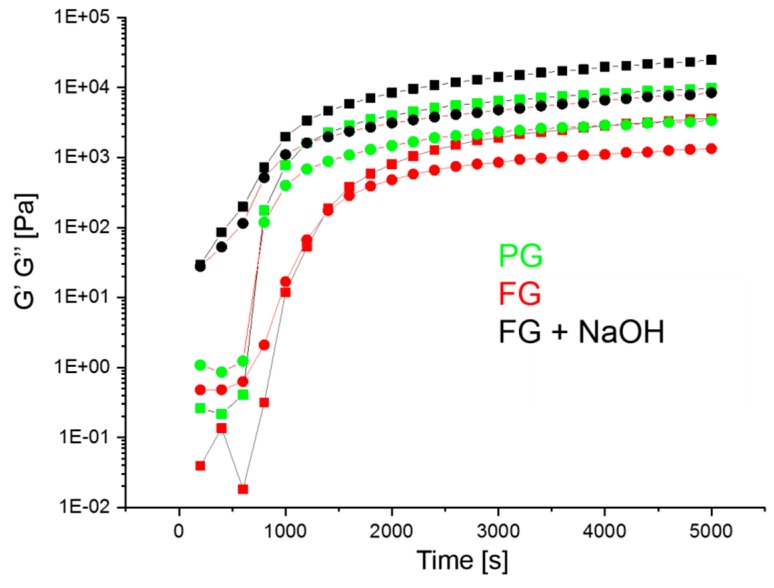
Time dependence of storage modulus G’ (square) and loss modulus G” (circle) of PG (green curves), FG (red curves), and FG+NaOH (black curves) solutions.

Given the importance of flow properties and viscoelastic behavior of the solution in the electrospinning process, FG solution composition was optimized on the basis of rheological measurements, taking as a reference a PG solution whose electrospinnability was previously demonstrated by some of the Authors [8]. Figure 1 reports the storage modulus (G′) and the loss modulus (G″) over 1 h 40 min period for the analyzed solutions. FG shows a rheological behavior similar to that of PG. Indeed, although FG moduli are slightly lower than those of PG, both solutions behave as viscoelastic liquids (G′ < G″) at initial times (up to 12 min for PG and up to 23 min for FG), then they switch to a solid-phase dominated behavior (G′ > G″), as demonstrated by the achievement of the cross-over, referred to as the “gel point” attributed to the increased molecular association. Finally, the moduli reach a fairly constant value. The presence of NaOH affects the viscoelastic properties of the solutions. Indeed, FG+NaOH presents higher values of G′ and G″ than FG, even higher than PG, and it shows no cross-over and a solid-phase dominated behavior since the beginning of the measurement. This result might indicate that crosslinking reactions due to citric acid are more likely to happen in the solution at pH 3.7 rather than in that at pH 1.8.

For all the solutions, even if a gel point was observed and the solid-like behavior dominated over time, the small differences between G′ and G″ indicate the formation of a weak gel that did not hinder electrospinnability of the solutions.

### 3.2. Characterization of Fish Gelatin Electrospun Mats

**Figure 2:**
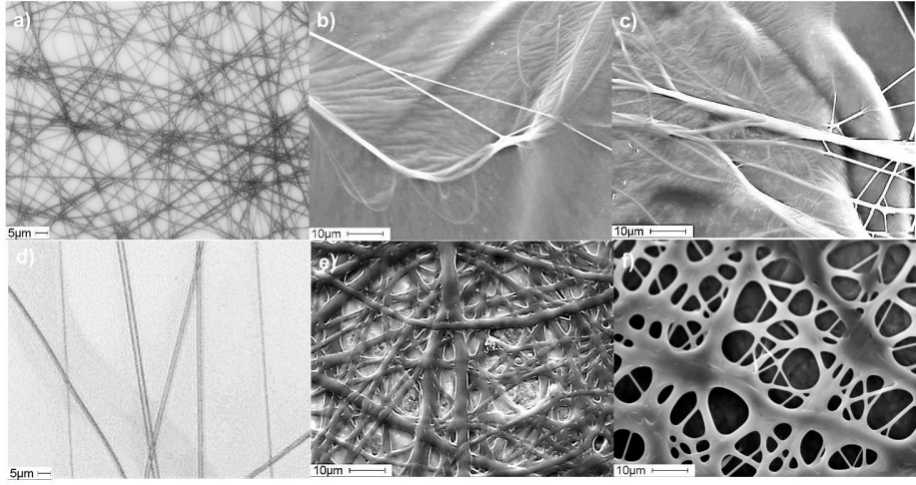
OM (**a**,**d**) and SEM images (**b**,**c**,**e**,**f**) of electrospun mats from FG (**a**,**b**,**c**) and FG+NaOH (**d**,**e**,**f**) solutions: mats as spun (**a**,**b**,**d**,**e**) and after the thermal treatment (**c**,**f**).

The OM and SEM images of the electrospun mats obtained from FG and FG+NaOH solutions are reported in Figure 2. Interestingly, the OM images (Figure 2a,d) show that bead-free and regular microfibers were obtained for both FG and FG+NaOH solutions. In contrast to previous findings [31], our results demonstrate that electrospinning of FG from a citric acid/water solution without the addition of acetic acid to form microfibers was possible. However, although both the solutions turned out to be electrospinnable, the fibrous morphology of mats obtained from FG solution was not preserved over time, since the mats turned into films, with a barely recognizable fibrous structure, in few hours, as demonstrated by the SEM micrographs (Figure 2b).

Addition of NaOH to FG solutions increased the pH values from 1.8 to 3.7 and improved the fiber stability over time. The obtained fibrous mat was able to preserve its morphology better than fibers obtained from FG solution (compare Figure 2b,e), although fiber fusion at their contact points could be noticed.

In agreement with previous literature findings [21,30], the thermal treatment performed on these mats immediately after their fabrication came out to get a beneficial effect on the resulting morphology, since the fibrous morphology of the mats is better maintained with respect to the as spun mats (Figure 2c,f). It is pointed out that, even if the fiber diameter was hardly measurable due to the many fusion points among fibers, after the thermal treatment a fiber diameter of 2.19 ± 0.07 µm and 4.42 ± 0.05 µm was evaluated for FG mats and FG+NaOH mats, respectively.

To assess the extent of the crosslinking reaction between gelatin and citric acid, the amount of ε-amino groups of gelatin reacted with citric acid was calculated. In line with the morphological results, only a small amount of ε-amino groups was crosslinked with citric acid in the mats obtained from FG solution, leading to a crosslinking extent of 12%. On increasing the pH up to 3.7 in the FG+NaOH solution, crosslinking extents of 25% and 38% were achieved for FG+NaOH mat as spun and thermally treated, respectively. These results are in agreement with previous findings [35], and highlight that the crosslinking reactions can take place also at room temperature in the water solution of FG with citric acid, even if an increase of temperature up to 80 °C is needed to speed up such crosslinking reaction.

On the basis of the above-described results, the following considerations can be drawn. For the mat obtained from FG solution, the obtained morphology is well explained by the low crosslinking extent of the fibers and it is mainly ascribed to the low pH of the solution, since at pH 1.8 the crosslinking reactions can difficultly take place. Moreover, the widely reported mechanism driving the crosslinking of proteins or molecules containing amino groups in the presence of citric acid lies in the formation of reactive citric anhydride from citric acid and in the nucleophilic substitution occurring between the carboxyl groups of the anhydride and the amino groups of the considered protein or molecule [21,34,35]. Since type-A fish gelatin is employed in this work and its isoelectric point is in the pH range of 6.0–9.5 [36], the protonation of the amine groups takes place in the strong acidic conditions of FG solutions (pH 1.8), thus limiting the crosslinking reaction. An increase of pH up to 3.7, even if lower than fish gelatin isoelectric point, would favor the deprotonation of –NH_3_^+^ groups in –NH_2_, and the above described nucleophilic substitution was more likely to occur with the formation of amide groups. The pH value of 3.7 was, thus, selected in order to achieve the best compromise between the number of amine groups available for crosslinking and the known crosslinking mechanism of citric acid, which has been reported to occur at the highest extent at pH 3.5 [21].

**Figure 3:**
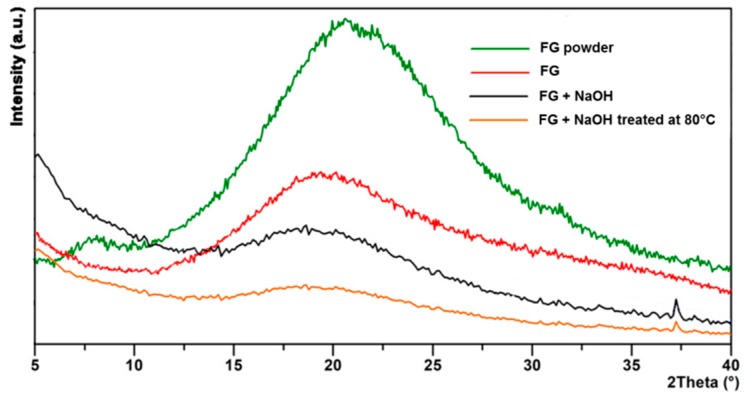
WAXD patterns of FG powder, FG, FG+NaOH, and thermal treated FG+NaOH electrospun mats.

The influence of the electrospinning process on structural properties of gelatin was investigated on fish gelatin powder and on the obtained mats through wide-angle WAXD analysis. In that way, the relative triple-helix content of fish gelatin materials was analyzed in detail (Figure 3). It is well known that the collagen WAXD pattern includes two broad diffraction bands. The first one, centered at about 8°, related to the triple helix diameter, while the second one at around 21° is related to the distance between amino acidic residues in the helix. These reflections are typically observed also in the pattern of partially renaturated gelatin powder and gelatin films [37].

In agreement with these data, results reported in Figure 3 show that fish gelatin powder exhibits the two reflections centered at about 8° and 21°, as previously reported [38,39]. However, reflection at 8° disappears after the electrospinning process of both FG and FG+NaOH solutions, as a consequence of acidic pH. This result can be explained considering that, as previously observed for gelatin solubilized in acetic acid solutions [8], citric acid prevents the gelatin’s partial renaturation which takes place during gelling from aqueous solution and a random coil conformation is favored, decreasing the number of single left-hand helix chains and residual triple-helix conformations. Furthermore, citric acid also influences the diffraction reflection located at about 21°, whose intensity decreases as a consequence of the decrease of the single left-hand helix chain content. Addition of NaOH to FG solution, with a consequent increase of pH from 1.8 to 3.7, does not change the diffraction pattern significantly, whereas the thermal treatment performed on FG+NaOH mat further decreases the broad band at about 21°.

**Figure 4:**
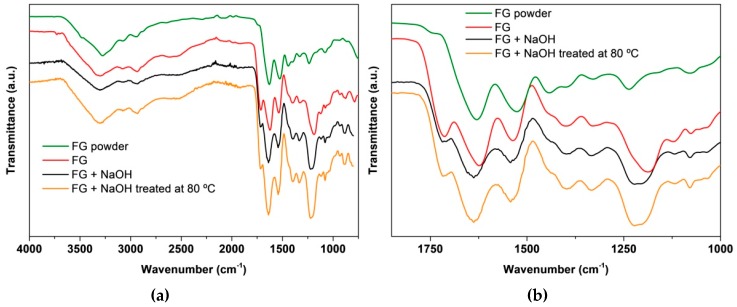
ATR-FTIR spectra of FG powder, FG, FG+NaOH, and thermally treated FG+NaOH electrospun mats (**a**) from 4000 to 800 cm^−1^ and (**b**) from 1850 to 1000 cm^−1^.

Figure 4 shows the ATR-FTIR spectra of the electrospun mats obtained from FG and FG+NaOH solutions before and after the thermal treatment, FG powder is also reported for the sake of comparison. The broad band above 3000 cm^−1^, observed in all spectra shown in Figure 4a, corresponds to the hydroxyl and amino groups [40]. Some changes can be observed in FTIR spectra, in particular, in those bands associated to the peptide bonds in gelatin: amide I (C=O stretching), amide II (N–H bending), and amide III (C–N stretching). As can be seen in Figure 4b), there was a shift of these bands to higher wavenumbers from FG mat to FG+NaOH mats, indicating that the citric acid incorporated into the formulation causes new interactions between the amino groups of gelatin and the carboxyl groups of citric acid [27,41], in accordance with the shift of the characteristic band related to the carboxyl group in citric acid from 1748 cm^−1^ to 1715 cm^−1^ [42].

**Figure 5:**
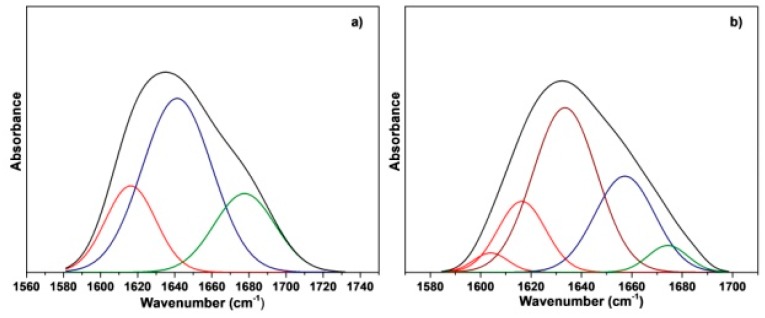

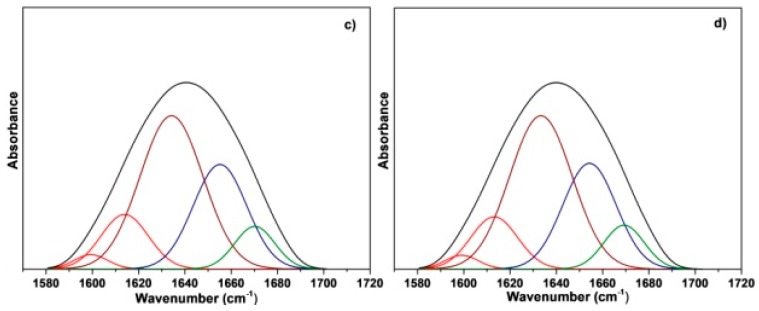
Curve fitting spectra of amide I band (black curve) for (**a**) FG powder, (**b**) FG, (**c**) FG+NaOH, and (**d**) thermal treated FG+NaOH electrospun mats (β-sheet conformation, red curve; random coil conformation, brown curve; α-helix conformation, blue curve; β-turn conformation, green curve).

The band corresponding to amide I is related to the secondary structure of the protein backbone and it is generally used for the quantitative analysis of the secondary structures. Hydrogen bonding plays a significant role in stabilization of protein secondary structure. Indeed, inter-peptide hydrogen bonding stabilizes secondary structures (i.e., α-helix and β-sheet conformations), while peptide-water hydrogen bonding competes against peptide bond-peptide bond hydrogen bonding. Due to the central role of hydrogen bonding in protein folding, the analysis of this band is of great importance (Figure 5).

The assignment of absorption peaks in amide I band is as follows: two peaks from 1603 to 1616 cm^−1^ support β-sheet conformation, the peak at 1634 cm^−1^ corresponds to random coil conformation, the peak from 1641 to 1650 cm^−1^ is associated to α-helix conformation, and the peak centered at 1670–1678 cm^−1^ is assigned to the β-turn conformation of the hairpin-folded antiparallel β-sheet structure (Table 1).

As shown in Figure 5 and Table 1, electrospinning causes changes in the secondary structure of gelatin. While FG powder shows α-helix conformation, a random coil is the predominant conformation for electrospun mats, in accordance with the results found by XRD, which show amorphous structure with no remaining triple helix structure.

## 4. Conclusions

An environmentally friendly chemical strategy to successfully electrospin fish gelatin from a solution containing only citric acid in an aqueous solution was demonstrated. Citric acid was used as a benign acid to solubilize gelatin and allow the electrospinning process and, at the same time, as a crosslinking agent. The pH of the spinning solutions turned out to have a strong influence on the viscoelastic behavior of the solutions, as well as on the crosslinking extent that, in turn, influenced the fiber morphology stability. Rheological measurements provided evidence that in all solutions the solid-like behavior dominated over time, but the weak gel formed did not hinder electrospinnability of the solution. The solution at pH 3.7, showing higher values of G′ and G″ moduli, was characterized by a higher extent of crosslinking with respect to the solution at pH 1.8. Although microfibers were obtained for both FG and FG+NaOH solutions, the increase of solution pH from 1.8 to 3.7 was necessary to maintain the fibrous morphology also in the mat. A subsequent thermal treatment at 80 °C of the electrospun mat turned out to significantly increase the morphological stability of the mat. The crosslinking degrees of the mats were in line with the morphological results (FG mat = 12%, FG+NaOH mat = 25%, thermally treated FG+NaOH mat = 38%). Gelatin denaturation after the electrospinning process was demonstrated by the absence of the diffraction band related to the triple helix diameter, as expected since gelatin solubilization in acidic solvents is known to prevent the partial renaturation of gelatin. ATR-FTIR characterization confirmed this result and demonstrated that the gelatin structure changed from α-helix to random coil conformation as a consequence of the electrospinning process. Although further studies are necessary to find the best solution composition to further optimize the final morphology as well as the crosslinking extent of the produced mats, this work is the first successful attempt to produce crosslinked electrospun fish gelatin fibers through the use of a citric acid/water solution.

## Figures and Tables

**Table 1:** Resulting percentage of the curve fitting of amide I for FG powder, FG, FG+NaOH, and thermal treated FG+NaOH electrospun mats.

Amide I Area	1603–1616 cm^−1^ (%)	1634 cm^−1^ (%)	1641–1650 cm^−1^ (%)	1670–1678 cm^−1^ (%)
FG powder	20.37	-	57.00	22.63
FG	18.75	48.73	26.54	5.98
FG+NaOH	16.94	44.70	29.79	8.57
FG+NaOH treated at 80 °C	16.16	44.29	30.84	8.71
